# What Key Aspects Do ICOs Reveal About Their Businesses?

**DOI:** 10.1007/978-3-030-58858-8_5

**Published:** 2020-08-18

**Authors:** Gabriella Laatikainen, Alexander Semenov, Yixin Zhang, Pekka Abrahamsson

**Affiliations:** 6 IT University of Copenhagen, Copenhagen, Denmark; 7 University of British Columbia, Vancouver, BC Canada; 8 Faculty of Information Technology, University of Jyväskylä, Jyväskylä, Finland; 9 Swedish Center for Digital Innovation, University of Gothenburg, Gothenburg, Sweden

**Keywords:** ICO, Blockhain, Business models

## Abstract

Blockchain technologies disrupt industries by enabling decentralized and transactional data sharing across a network of untrusted participants, among others. Initial Coin Offerings (ICOs) are a novel form of crowdfunding through which hundreds of blockchain-enabled businesses manage to raise billions of dollars in total only in United States. However, there is a lack of understanding of the ICO phenomenon especially related to the business aspects. In this paper, we describe the results of an exploratory study of 91 ICOs and identify the key business model elements that ICOs reveal in their websites and whitepapers. Furthermore, we also note the immaturity and lack of transparency of the business aspects of businesses behind the ICO campaigns.

## Introduction

The emergence of blockchain technologies sparked a lot of interest towards the blockchain technology. Initial Coin Offerings (ICOs) represent an unregulated fundraising model that enable blockchain-based projects to be funded via crowdfunding model [1, 2]. To date, the most successful ICO, Filecoin was able to collect more than 257 million USD while the average ICO raised a total of almost $11.4 billion in 2019 [3]. It has to be noted that besides providing an easy opportunity to gain great profits, ICOs are often frauds that fund scams.

ICOs are poorly understood and there is a need for more research in this area [4, 5]. Earlier research discusses how blockchain technology impacts the business models of existing companies using case study methodology and literature review (e.g. [6, 7]). However, research is still nascent related to the means of possible ICO investors to get an overview of how the business behind the ICO makes money (i.e., the business model). Thus, this study focuses on blockchain-enabled business models and aims to answer the following research question: *What business model characteristics do blockchain*-*enabled businesses reveal in their ICO’s websites and whitepapers?* To answer this question, we collected a sample of 91 ICOs from 14 ICO enlisting sites and studied the business model aspects of these ICOs through content analysis. This study contributes to the growing body of blockchain literature by identifying the revealed business model elements of blockchain-enabled businesses that aim to raise funds through ICOs. These insights can be used both in further research and in practice.

## Related Work

A firm’s business model is a conceptual model of a business: a description of how a company organizes itself, operates, and creates value [8, 9]. It is an abstract concept; it can be seen as a representation of a company or as a tool that provides a picture of a firm’s competitiveness [10]. Business models are often viewed from a component-oriented perspective. Despite the business models’ importance, researchers have not formed a consensus regarding either the core components of business models or their level of abstraction [11]. However, most researchers agree that business models contain the following key components: (1) *value proposition* (i.e., the product and/or service portfolio), (2) *revenue logic* (i.e., a top-level description of a business’s revenue sources) and (3) *activities* (i.e., actions in order to create and deliver values to customers) [8, 10–12].

Blockchain can be seen as a distributed database that maintains a continuously growing list of records linked to each other [13]. Blockchain database is secure by design, and once the block is recorded there, it cannot be modified retroactively. Blockchain relies on a peer to peer (p2p) network without any central coordinating node. Technically, each transaction contains a small piece of code that allows complex cryptographic validation of transactions. This code presents a smart contract that is defined as “complex application involving having digital assets being directly controlled by a piece of code implementing arbitrary rules” [14]. Thus, these self-executing digital contracts (i.e. smart contracts) and intelligent assets that are controllable through internet (i.e., smart properties) enable the emergence of new types of businesses where organizations operate in a network with limited or no human interactions [15].

Blockchain technologies affect the business models in different ways, such as by authenticating traded goods, via disintermediation and via lowering transaction costs [7]. Related to the value proposition, Morkunas et al. found that blockchain technology can provide verifiability, access to new products and services, faster or less expensive transactions, and fewer middle layers [6]. The greatest revenue stream for blockchain-enabled businesses is the possibility to raise funds through ICOs; however, other revenue sources are transaction fees and service or platform fees [6]. Key activities include transforming the business processes and the key resource is the peer-to-peer network [6].

## Methodology

We studied the business aspects of 91 ICOs in May and June 2018 in order to identify the special characteristics of blockchain-enabled business models. In the next subsection we describe the data sample and the data collection. Then, we overview the details of the data analysis.

### Data Sample

To ease the process of the data collection, we run crawling scripts that collected the ICOs name, link and category from each ICO listing sites. We aimed to crawl all the sites whose primary task is to enlist ICOs and we used Google search to identify these. After some exploratory exercises, the crawling scripts for 14 websites were running on the same day. We gathered the data from the following websites: Bestcoins, Coingecko, Coinmarketplus, Icohotlist, the tokener, icorating, icomap, topicohotlist, coinschedule, icowhatchlist, icotracker, icobazaat, listico and icobench.

After identifying the sample frame, we eliminated the duplicates and the resulting dataset contained 4127 ICOs. Then, we grouped the data by the ICOs categories^1^. As a final step, we identified the final data sample by choosing randomly at least three ICOs from each category in order to increase the industry coverage of the sample data.

### Content Analysis

In the data analysis phase, we used content analysis [16]. First, we manually collected information on the sample ICOs’ business models. We used the ICOs websites, the whitepapers and executive summaries as well as data on the ICO listing sites as data sources. In the first phase of the content analysis, we identified the characteristics of the ICOs business models. The gathered information included more than 30 business model characteristics, out of which many aspect could not be found for many ICOs. In the second phase, we clustered homogeneous elements and identified the business model aspects that are special for blockchain-enabled businesses.

Second, we calculated the descriptive statistics. Some of the characteristics could not be find in case of every ICO. In the calculations, these missing values were not taken into account, i.e. the percentages were calculated so that 100% is the number of ICOs with available data.

During our empirical analysis, we paid special attention to the reliability and validity of the study in each step. First, the listing sites were identified and discussed by two authors. Second, the content analysis were carried out by three authors. In order to avoid coder bias, we cross-validated the results and some of the ICOs were coded by two different persons. In these cases, the results were compared and discussed and the differences were negligible.

## Findings

Our study revealed that after a two-month period, only 84% of the ICOs websites were active and only 72% after two years. Furthermore, based on our findings, even though the quality of the whitepapers differed significantly, in general, the amount of information about the ICOs strategy, vision and operations was rather limited. The whitepapers aimed to describe the ICO’s goals and motivation, the underlying blockchain technology, the details of the ICO’s financial roadmap, the target customer segments, the key partners, the risks, etc. However, most of the whitepapers lacked some of the information, they were not transparent and not detailed enough. One of the common problems was that they did not contain clear financial roadmap or information on detailed risk assessment. However, in what follows, we describe the common patterns on different aspects of the ICO’s business models.

### The Value Proposition

The ICOs described the value proposition in their whitepaper (i.e., the blockchain-enabled service that they developed) through two common aspects: the service characteristics and the benefits of blockchain technology. It has to be noted, that in some cases, the advantages of blockchain-based service as compared to a similar service that uses alternative technologies remained unclear.

#### Service Characteristics.

One of the important aspects of the service that they develop is the underlying blockchain *platform* that they build their solution on. Our results showed that the most used platform was the Ethereum platform and the tokens were ERC20 tokens. In our sample, 89% of the ICOs used Ethereum as a base platform. The second most used platform in our sample data was Bitcoin, followed by Waves, Lisk, MultiChain, Pivx, etc.

Some of the ICOs do not use *public* blockchain, but they build their own *private* network. That is, instead of allowing everyone to participate in the network, and encouraging more participants to join, joining a private blockchain requires an invitation and complying with a set of rules. This restriction on the participants can be regulated by existing nodes, a regulatory authority or a consortium. The greatest advantage of using a private blockchain as compared to the public one is that it requires less computational power due to the smaller number of nodes and it assures higher level of privacy and security requirements. In our sample, there was only one ICO whose value proposition was based on a private blockchain.

#### Benefits of Blockchain Technology.

Based on the sample data, the value of the offering was mainly described through the benefits of blockchain technology.

First, ICOs promised to provide a quality service that was more secure and confident because of the use of blockchain technology. For example, the ICO Adamant provided a private messenger platform where the blockchain technology ensured that no one had access to private data except of the owner.

Second, businesses could take use of the transparency and resistance to data manipulation that the blockchain technology provided. For example, the ICO Affchain offered a marketing protocol and a marketplace where businesses and affiliates met and made deals in online advertising. The ICO provided a cryptographically verifiable value distribution mode that increased the level of trust and enabled cost-reduction for the parties because of automatic verification without human interactions.

Third, businesses could offer cost reduction because of the distributed network where intermediaries were not needed. In blockchain-enabled businesses users could find and contact each other directly that lead to reduced transaction costs. For example, the ICO Lotuscore provided a game platform and the players were able to trade games with friends while the developers earned 100% of the revenue derived from selling their digital game.

Forth, blockchain-enabled services could provide more power for the users. In a blockchain-enabled network there was no centralized influence and thus, the control was distributed. For example, the ICO OneRoot proposed a platform that enabled building relationships based on equal co-operation and common development instead of an ecosystem with centralized entities.

Fifth, blockchain-enabled services benefitted from the self-executing smart contracts where no or limited human interactions were needed to ensure different processes. This simplified work processes and thus, it caused cost-reduction. For example, the ICO VLux proposed a renewable energy trading solution that promised cost reduction due to trading at optimal times.

Sixth, in some cases, blockchain-enabled services allowed users to pay anonymously in order to ensure their privacy. For example, the ICO Clean SL8 provided a communication platform for life coaching where the users valued privacy and the ability to stay anonymous.

Seventh, blockchain-enabled services provided the possibility of using cryptocurrencies as payments for the ICOs service. For example, in an ICO project Lunar, the founders proposed online dating services. Users needed to pay in cryptocurrency in order to communicate with other users.

Eighth, some of the value proposition of ICOs were unique because they were related to blockchain technologies or the cryptocurrencies. Indeed, new businesses emerged that addressed the possible investors’ interest in blockchain. For example, the ICO Takeprofit provided a platform where experienced traders offered their advice on cryptography investments for inexperienced users.

### Revenue Logic

In this study, we found that in most cases, the revenue logic of ICOs towards the customers was not well described. The ICOs gave some hints on the ways that the business might bring profits; however, the detailed concrete description on the revenue sources and the magnitude of the revenues was frequently missing in the sample we collected.

As a general note, the results of this study showed that the commonly used pricing models did not differ significantly from the businesses that offered a similar service without the use of blockchain technology. In other words, the pricing models depended mostly on the type of the service that the ICOs offered. The most used pricing models in our sample were the following: (1) *pay*-*per*-*use* (customers are charged a so-called transaction fee each time they use the service), (ii) *advertisement model* (customers use the service for free and advertisers pay for their advertisements that are shown to the users), (iii) *micropayments* (In ICOs that provide gaming platforms, micropayments are used to enhance the players’ user experience) and (iv) *price discrimination* (different prices are charged depending on customers’ characteristics, such as financial status or country [17]).

### Activities

The activities that ICOs promised to perform could be investigated from the data on the usage of funds that described the activities and other cost factors that the collected money would be used for. In order to create and deliver values to their customers, the ICOs had to develop their service, market it to the customers, provide maintenance and administrative services, solve legal conflicts, and so on.

One of the special characteristics of blockchain-enabled business model is the importance of the ecosystem around the service where the actors jointly create value. One example could be the *bounty* program that ICOs provide to incite investors to perform small tasks and gain some reward (usually in form of tokens) in return. The bounty tasks vary greatly among ICOs; they can be related to marketing, bug reporting, development, promotion, translation, proofreading, website design, etc. We found that 60% of our sampled ICOs used bounties.

Another example of value co-creation is the use of *referral program* as a channel through which customers are reached and targeted [18]. ICOs typically offer the investors the possibility to gain tokens by advertising the ICO to their friends and family, or through their websites or different social media sites. The ICO benefits from this program in three ways. First, due to network effect, the value of their service increases as the number of users increase. Second, the word-of-mouth builds trust in new customers. Third, the program brings cost reduction by decreasing the marketing and advertising costs. In our sample data, 90% of the ICOs had some kind of loyalty program.

## Discussion and Conclusions

In this study we took a sample of 4127 ICOs collected from 14 ICO enlisting sites and investigated the business model aspects of 91 ICOs by analyzing the ICO enlisting websites, the ICOs’ websites and their whitepapers. We found that the amount of available information on the ICOs business models was rather limited. That is, the websites and the whitepapers lacked important details and concrete data on the business strategy. However, our study found common patterns that blockchain-enabled businesses revealed about their business models in the whitepapers of their ICO.

In Table 1, the business model aspects of blockchain-enabled businesses are summarized. The findings revealed that ICOs built their value proposition on the benefits of the blockchain technology, such as security, transparency, confidentiality. In blockchain-enabled businesses middlemen were not needed, and this might cause cost reduction. The self-executing smart contracts simplified processes because they eliminated or mitigated the need for human interactions. Furthermore, in a blockchain-enabled service different cryptocurrencies could be used for payments.

**Table 1:** Blockchain-enabled business models aspects revealed from ICOs’ whitepapers

Value proposition	*Service and platform characteristics*: Ethereum (89% of the ICOs), Bitcoin, Waves, Lisk, MultiChain, Pivx *Technology*-*enabled benefits*: security, transparency, confidentiality, no intermediaries, simplified processes, possible cost reduction, using cryptocurrencies as payments, possibility to stay anonymous
Revenue logic	Common revenue models include pay-per-use, advertisements, micropayments
Activities	Service development, marketing, maintenance and administrative services, solving legal conflicts, implementing referral programs and bounties (60% of the sample had bounties)

The revenue logic towards the customers was based on the type of the service rather than on blockchain characteristics. For example, game platforms used micropayments while electronic marketplaces got their revenues from advertisements.

In blockchain-enabled businesses, the actors of the ecosystem (e.g. investors, users) were involved in different activities that the service required to create and deliver value. For example, the referral programs provided incentives for the actors to advertise the service to their friends, family and other individuals. Besides, everybody could perform specific tasks related to development and marketing of the service and get some rewards in return.

This study found that only 84% of the ICOs websites were active after a two-months period and only 72% were active after two years. The reasons for an ICO not to continue its business can vary. First, if it does not get the amount of funds required for the development of the service (Soft Cap), then the money can be returned to the investors and stop the business. Second, there is no guarantee that ICOs give the investors’ money back. Thus, it can happen, that ICOs shut down their websites and take the money they received as funds without any consequences. This is the consequence of the unregulated environment where ICOs are not bound to economic regulations and laws and thus, ICOs provide easy means for frauds.

This study is an exploratory study that has some limitations. First, the sample was collected in a limited period of time that had a limitation on the generalizability of the results because of the fast changes of the market. Second, some of the information on the websites and in the whitepapers were changed after the two-month period that the empirical study was carried out. Furthermore, the available information was not concrete and detailed enough. Thus, there is a need for additional research in this area that allows us to compare the results and give us further insights. For example, the ICOs studied in this research could be analyzed again to investigate how their business model changed based on longitudinal data.

## Footnotes

1 We grouped our data into the following ICO categories: Internet, Tourism, Cryptocurrency, Business services, Platform, Retail, Investment, Infrastructure, Financial services, Trading, Entertainment, Casino Gambling, Energy, Smart contract, Manufacturing, Media, Communication, Banking, Charity, Virtual Reality, Electronics, Software, Business services, Data analytics, Sports, Real estate and Health.
